# Bamboo shoot fiber prevents obesity in mice by modulating the gut microbiota

**DOI:** 10.1038/srep32953

**Published:** 2016-09-07

**Authors:** Xiufen Li, Juan Guo, Kailong Ji, Ping Zhang

**Affiliations:** 1 Key Laboratory of Tropical Plant Resources and Sustainable Use, Xishuangbanna Tropical Botanical Garden, Chinese Academy of Sciences, Menglun, Mengla, Yunnan 666303, China; 2 University of Chinese Academy of Sciences, Beijing 100049, China

## Abstract

Dietary fiber has been shown to prevent high-fat diet induced obesity through modulating the gut microbiota; however, quality difference in fiber type is largely unknown. We performed a 6 week study on C57BL/6J mice fed a macronutrient matched high-fat diet with different fiber types including cellulose (HFC), bamboo shoot fiber (HFBS) and several other commonly consumed fibers. Our results showed that the HFBS group exhibited the lowest weight gain among all diet groups and had improved lipid profiles and glycemic control compared with the HFC group. As revealed by 16S rRNA gene sequencing, loss of diversity in the gut microbiota induced by the HFC diet was largely prevented by the HFBS diet. Moreover, compared with the HFC diet, the HFBS diet resulted in markedly increased relative abundance of *Bacteroidetes* and strong inhibition of *Verrucomicrobia*, two divisions strongly correlated with body weight. In conclusion, the present study provides evidence of a quality difference among different types of dietary fibers and shows that bamboo shoot fiber is the most effective in suppressing high-fat diet induced obesity. Our findings indicate that bamboo shoot fiber is a potential prebiotic fiber which modulates the gut microbiota and improves host metabolism.

Prevalence of obesity and overweight has risen substantially in the past three decades in China as in other regions of the world. In 2013, the absolute number of obese people in China was exceeded only by that in the USA^1^. The huge burden on society associated with treatment of obesity related diseases demands urgently for effective methods for managing this public health problem^2^,^3^. Dietary and lifestyle changes remain the fundamental strategy for the prevention and therapeutics for obesity and related metabolic disorders^4^. High consumption of dietary fiber has long been associated with reduced risk of cardiovascular disease, diabetes, obesity and gastrointestinal disorders^5^,^6^,^7^,^8^. However, mechanisms remain poorly understood and there are still controversies to explain the effectiveness of different types of dietary fibers^9^,^10^.

In recent years, knowledge about the role of the gut microbiota in health has grown tremendously. The gut microbiota, composed of trillions of microorganisms, is not our “forgotten organ”^11^ anymore and has been accepted as an integrative part of our metabolome in a “super-organismal” context^12^. The microbiota plays a major role in health and disease by shaping our immune system and metabolism^13^. Altered microbiota has been associated with obesity and diabetes in human subjects^14^,^15^,^16^. Genetically obese mice are found to harbor an intestinal microbiota which promotes energy harvest^12^. Diet-induced obesity is shown to be linked to reversible alteration in the mouse distal gut microbiome^17^. These findings suggest that the gut microbiota may be a target for both prevention and therapeutics of obesity and metabolic syndrome.

The essential role of dietary fiber in maintaining body weight and overall health has been increasingly recognized so that a dietary recommendation of >25 g per day for healthy adults is made by WHO^18^. The definition of dietary fiber has evolved in the last 30 years but the same basis remains, as carbohydrate polymers that are resistant to digestion and absorption in the human small intestine, whether they are natural or synthetic, soluble or insoluble, fermented or not fermented, viscous or non viscous^19^. Recent studies demonstrated that several fibers, including wheat bran-derived alkylresorcinols^20^, chitin-glucan fiber^21^, and hydroxypropyl methylcellulose^22^ suppressed body weight and improved host metabolic disorders in high-fat diet induced obese mice through counteracting gut dysbiosis. The common feature of these studies is that they investigated the effects of additional amount of a specific fiber in a high-fat diet, i.e. the quantitative effects of dietary fiber on physiology, and highlighted the mechanism of prebiotic effects of insoluble dietary fiber to explain the benefits for obesity related disorders. It is well known that various types of dietary fibers differ greatly in physiochemical properties and thus may vary greatly in their physiological effects^23^,^24^. However, there have been very limited studies to compare their difference in influencing host metabolism. For example, inulin was reported to reduce body weight and improve metabolic disorders when added to a high-fat diet^25^. However, when the same amount of inulin and cellulose was used in a high-fat diet, inulin did not show benefits^26^. Although soluble fibers were reported to lower cholesterol levels and reduce diabetes risk in short term studies^6^, comparison of the qualitative difference between guar gum and insoluble oat fiber side by side showed that in the long term, soluble fiber promoted obesity and fermentation contributed to more body weight gain^27^. Therefore, more studies are needed to delineate the quality difference of fiber type on host physiology.

Bamboo shoots promote health in many aspects including improving digestion, relieving hypertension, and preventing cardiovascular disease and cancer, according to records in ancient Chinese literature in medicine^28^. The health-promoting effects of bamboo shoots may be due to their high content of dietary fiber. Higher than most commonly consumed vegetables, the fiber content of bamboo shoots can be around 40% of dry weight^29^. There have been very limited studies to characterize properties of the bamboo shoot fiber, especially its physiological effects. We observed that dietary fiber isolated from bamboo shoot *Dendrocalamus hamiltonii* exhibited extraordinary water and oil binding capacities as well as cholesterol and bile acid binding capacities (China patent No. 2015108901176), suggesting that bamboo shoot fiber may have a great impact on host metabolism and the gut microbiota.

Therefore, in the present study, we investigated in C57BL/6J mice whether high-fat diets with the same amounts of microcrystal cellulose and bamboo shoot fiber differentially affect body weight, glycemic control, lipid metabolism, as well as gut microbiota. For comparison purpose, we also included diet groups supplemented with wheat or soybean fibers which are two widely consumed insoluble fibers in China, or with inulin, which is a typical soluble fiber, or with mixes of bamboo shoot fiber and inulin in three ratios. Our goal is to compare the quality difference among several dietary fibers and identify one fiber or a fiber mix of insoluble and soluble fiber which is the most effective in suppressing high-fat diet induced obesity and obesity related metabolic disorders in mice and to assess whether the possible benefits are associated with changes in the gut microbiota.

## Results

### Bamboo shoot fiber was more effective in suppressing body weight gain on a high-fat diet than other fibers

**Figure 1:**
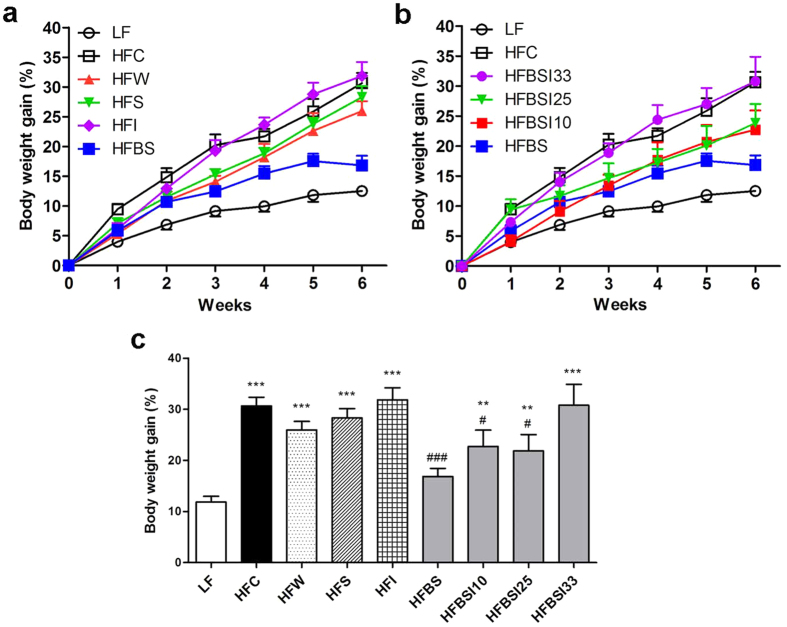
Bamboo shoot fiber prevented body weight gain in C57 BL/6J mice fed a high-fat diet for 6 weeks. Evolution of body weight gain of mice on (**a**) different dietary fibers, (**b**) or fiber mixes with different proportions of bamboo shoot fiber with inulin. (**c**) Relative body weight gain at week 6. Data are expressed as the percentage of body weight gain relative to the initial body weight (%). LF, low fat diet; High-fat diets with 10% fiber: cellulose (HFC), wheat fiber (HFW), soybean fiber (HFS), inulin (HFI), bamboo shoot fiber (HFBS), and mixed bamboo shoot fiber and inulin with ratios of 2:1 (HFBSI33), 3:1 (HFBSI25), and 9:1 (HFBSI10). Data are expressed as mean ± SEM of fifteen mice. **p < 0.01, ***p < 0.001, vs. mice fed LF diet. ^**#**^p < 0.05, ^**###**^p < 0.001, vs. mice fed HFC diet.

We first compared the effect of fiber type on prevention of high-fat diet induced obesity. The composition of the high-fat diets was comparable, with the only difference in fiber type (see Supplementary Table S1), and the diet with 10% cellulose (HFC) was considered as a high-fat diet control. A control low fat diet (LF) contained 4.4% fat and 5% cellulose. The purified bamboo shoot fiber consisted of 74.5% total dietary fiber (73.4% insoluble and 1.1% soluble) and 22.5% other components such as protein and ash. At the baseline, no significant difference in body weight was observed among all diet groups. After 6 weeks, in mice fed the HFC diet, body weight gained 31% of the initial value, whereas the mice fed the LF diet only gained 12.5% (Fig. 1a,c). Mice fed inulin, wheat, or soybean fiber exhibited no difference in body weight gain from the HFC mice. Only the mice fed the bamboo shoot fiber diet (HFBS) gained considerably less body weight than the HFC fed mice and did not show a significant difference from the mice fed the LF diet (Fig. 1a). To further test the difference of insoluble and soluble fiber in controlling body weight gain, we also compared three more high-fat diets with mixes of bamboo shoot fiber and inulin with ratios of 9 to 1 (HFBSI10), 3 to 1 (HFBSI25), and 2 to 1 (HFBSI33) respectively. The reason that different ratios were chosen is because dietary fiber provided by mixed diets which consists of two-thirds to three quarters insoluble fiber is recommended for maintaining health^30^. The mice on the HFBSI10 and HFBSI25 diets gained more body weight than the HFBS group, but still weighed significantly less than the HFC group (Fig. 1b,c). The mice on the HFBSI33 diet gained a similar weight to those on the HFC and HFI diets, adding another piece of evidence that inulin promotes weight gain and that an increased amount of inulin in a high-fat diet will attenuate the obesity-suppression effect of bamboo shoot fiber.

### Bamboo shoot fiber suppressed fat mass development and hyperplasia of adipocytes

**Figure 2:**
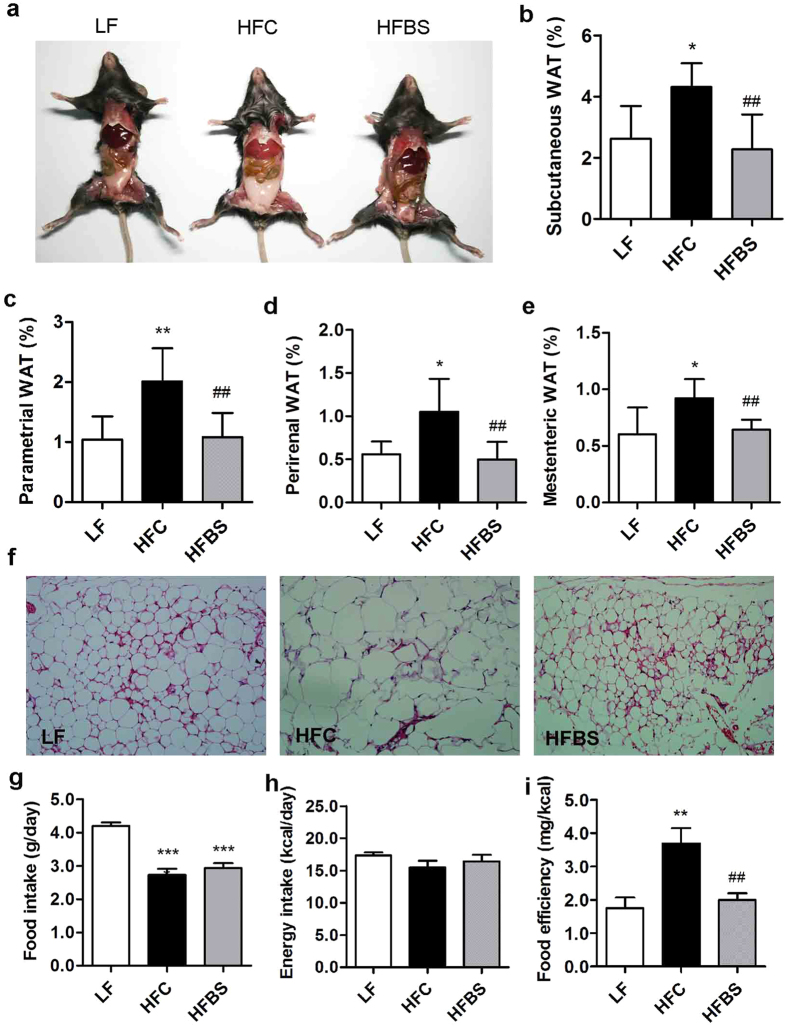
Bamboo shoot fiber reduced fat mass accumulation in high-fat diet fed mice. (**a**) Representative photographs of mice. The weight of white adipose tissue (WAT): (**b**) subcutaneous, (**c**) parametrical, (**d**) perirenal, and (**e**) mesenteric. (**f**) Adipocyte morphology was assessed by H&E staining (200×). (**g**) Daily food intake. (**h**) Daily energy intake. (**i**) Food efficiency. LF, low fat diet; HFC, high-fat diet with cellulose; HFBS, high-fat diet with bamboo shoot fiber. Data are expressed as mean ± SEM (eight mice for each group). *p < 0.05, **p < 0.01, ***p < 0.001, vs. mice fed LF diet. ^**##**^p < 0.01, ^**###**^p < 0.001, vs. mice fed HFC diet.

Because bamboo shoot fiber proved to be the best fiber in suppressing high-fat diet induced body weight gain, we focused on bamboo shoot fiber for further investigation. At the end of 6 weeks feeding, compared with cellulose, bamboo shoot fiber not only decreased body weight gain by 47% (Fig. 1c) but also decreased fat mass (Fig. 2). The HFBS mice showed decreased weight of typical adipose tissue depots including subcutaneous by 47%, parametrial by 46%, perirenal by 52%, and mesenteric by 30% (Fig. 2b–e). Body weight gain was found to be significantly correlated with the weight of the abdominal fat pad (r = 0.938, p < 0.01, Supplementary Figure S1), suggesting body weight gain was mainly due to lipid accumulation in the adipose tissue. No significant difference in the average food intake was observed in the HFC and HFBS groups (Fig. 2g), both with much lower values than that in the LF group. Energy intake was comparable among all three groups (Fig. 2h). However, food efficiency was significantly lower in the HFBS group than that in the HFC group, with values comparable to that in the LF group (Fig. 2i).

Histological analysis of white adipose tissue (WAT) revealed that HFC diet fed mice have larger adipocytes relative to LF diet fed mice. The HFBS mice exhibited a substantial decrease in the overall size of the adipocytes (Fig. 2f). High-fat diet induced obesity is known to be strongly associated with leptin, which is an adipokine secreted from WAT. Circulating plasma levels of leptin in the HFC mice were about 2-fold higher than those in the LF mice (793 vs. 469 pg/ml, p < 0.001, Table 1), whereas the HFBS mice showed decreased plasma leptin levels by 16.5% compared with the HFC mice.

Obesity is characterized with increased expression of genes involved in lipid metabolism and inflammation, so we also examined several obesity-associated genes, including TLR-4, GPR40 and 43, Fabp1, Scd1, and Mttp. However, bamboo shoot fiber did not affect their expression in WAT compared with cellulose (Supplementary Figure S2).

### Bamboo shoot fiber improved glycemic control and dyslipidemia in high-fat diet fed mice

The HFC mice exhibited elevated levels of fasting glucose, insulin and increased values of HOMA-IR compared with the LF mice (Table 1). The HFBS mice showed markedly decreased fasting glucose by 43%, decreased fasting insulin by 45%, and decreased HOMA-IR compared with the HFC group, restoring the values to the same levels as that in the LF group (Table 1). A glucose tolerance test was performed at week 5 of the experiment and glucose tolerance was quantified as the area-under the curve (AUC) integrated from 0–120 min. The HFC mice exhibited slightly impaired glucose intolerance with higher glucose levels at time points from 15 min to 120 min compared with the LF mice. Compared to the LF mice, the HFBS mice exhibited the same glucose level at 15 and 120 min time points and higher values at 30 and 60 min time points (Supplementary Figure S3). Glucose AUC was suppressed by 18% in the HFBS mice compared with the HFC mice (Table 1), suggesting better glycemic control than the HFC mice.

**Figure 3:**
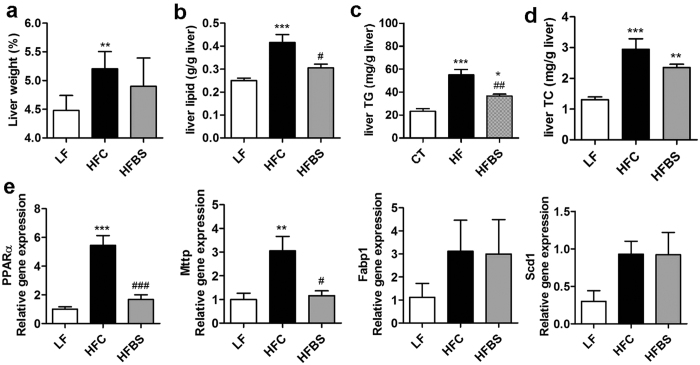
Bamboo shoot fiber prevented hepatic lipid accumulation. (**a**) Liver weight (% of body weight). (**b**) Lipid content in liver. (**c**) Hepatic TG. (**d**) Hepatic TC. (**e**) Expression of lipid metabolism related genes (PPARα, Mttp, Fabp1 and Scd1) in liver. Data are expressed as mean ± SEM (eight mice for each group). *p < 0.05, **p < 0.01, ***p < 0.001, vs. mice fed LF diet. ^**#**^p < 0.05, ^**##**^p < 0.01, ^**###**^p < 0.001, vs. mice fed HFC diet. LF, low fat diet; HFC, high-fat diet with cellulose; HFBS, high-fat diet with bamboo shoot fiber.

The HFC mice also exhibited hyperlipidemia as evidenced by increased levels of plasma triglycerides and cholesterol (Table 1). The HFBS mice exhibited significantly decreased plasma TC, HDL-C, and LDL-C compared with the HFC mice. In addition, high-fat feeding induced an increase in liver weight and lipid contents (Fig. 3a–d). Hepatic TG and TC were significantly reduced in the HFBS group compared with the HFC group (Fig. 3c,d). Furthermore, the HFC diet increased expression of several genes involved with regulating lipid metabolism, including PPARα, Mttp, Fabp1, and Scd-1. Bamboo shoot fiber completely suppressed the increase in PPARα and Mttp expression but had no effect on Fabp1 and Scd-1 expression compared with cellulose (Fig. 3e).

### Bamboo shoot fiber modulated the gut microbiota

**Figure 4:**
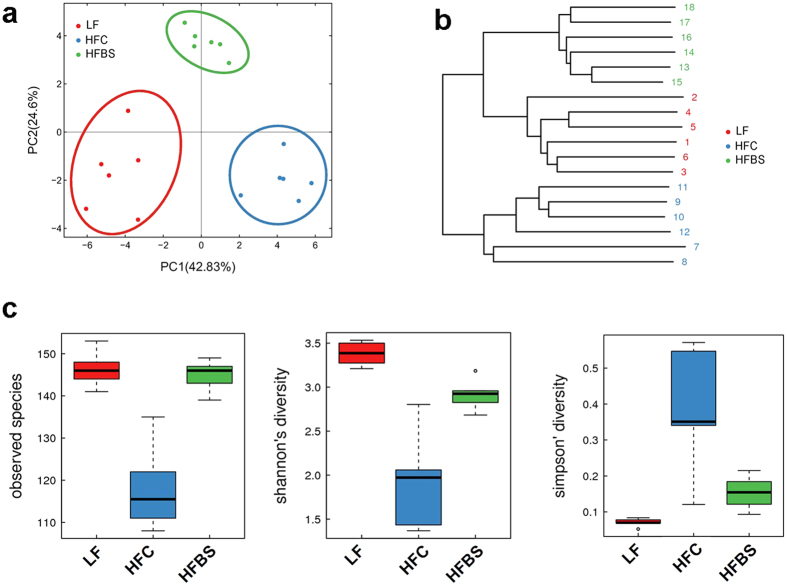
Bamboo shoot fiber modulated the structure and diversity of the gut microbiota. (**a**) Principal coordinate analysis (PCoA) and (**b**) Sample clustering results of the unweighted UniFrac distances of microbial 16S rRNA sequences from the V3-V4 region in cecal contents at week 6. (**c**) Alpha diversity analysis at the OTU level calculated on denoised sequences of mouse cecal microbiota. Six mice for each group. LF, low fat diet; HFC, high-fat diet with cellulose; HFBS, high-fat diet with bamboo shoot fiber.

To assess changes in the gut microbial community induced by bamboo shoot fiber, 16S ribosomal RNA gene from variable regions V3–V4 of the cecal samples from LF, HFC and HFBS diet groups were sequenced by Illumina HiSeq/MiSeq platforms and the results were presented as operational taxonomic units (OTUs) using a 97% homology cutoff value (Fig. 4).

The phylogenetic differences within the intestinal microbiota were assessed by Principal Coordinate Analysis (PCoA) analysis (Fig. 4a). The HFC diet fed mice had a distinct microbiota composition that clustered separately from the LF mice. The HFBS mice formed a distinct cluster from the HFC and LF mice. Hierarchical clustering showed that the microbial communities in the ceca of the HFBS mice were related more closely to those of the LF mice than those of the HFC mice (Fig. 4b). To assess intestinal microbial community structure, richness and evenness were calculated (Fig. 4c). At the OTU level, the HFC mice had decreased number of species compared with the LF mice. The HFBS mice exhibited a similar number of species as the LF mice. Diversity measured by Shannon’s richness index decreased significantly in the HFC group compared with the LF group. The HFBS mice had increased values of Shannon’s index compared with the HFC group, but did not reach the same level as the LF group. Diversity measured by Simpson’s evenness index also showed increased values in the HFC group compared with the LF group. The values of Simpson’s index in the HFBS group were lower than in the HFC group, but did not reach the same level as in the LF group (Fig. 4c).

**Figure 5:**
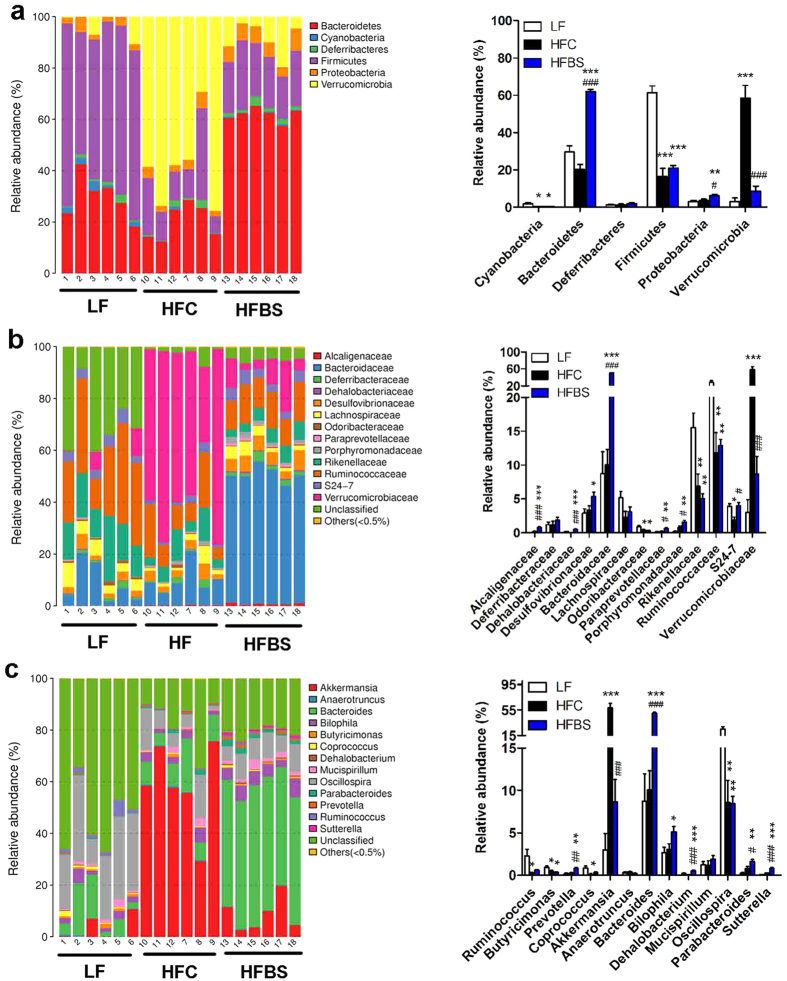
Bamboo shoot fiber modulated the composition of the gut microbiota. (**a**) Phylum-level, (**b**) family-level, and (**c**) genus-level taxonomic distributions of the microbial communities in cecal contents determined by next generation sequencing. Data are expressed as mean ± SEM (six mice for each group). LF, low fat diet; HFC, high-fat diet with cellulose; HFBS, high-fat diet with bamboo shoot fiber. *p < 0.05, **p < 0.01, ***p < 0.001, vs. mice fed LF diet. ^**#**^p < 0.05, ^**##**^p < 0.01, ^**###**^p < 0.001, vs. mice fed HFC diet.

To assess specific changes in the gut microbiota, we compared the relative abundance of the predominant taxa identified from sequencing in the three diet groups (Fig. 5). Significant differences in the composition of the gut microbiota were found at all taxonomic levels (Supplementary Figures S4 and S5). At the phylum level, the HFC diet associated cecal community showed a dramatic decrease in the relative abundance of *Firmicutes* and *Cyanobacteria* (p < 0.05) and a trend of decrease in the relative abundance of *Bacteroidetes* (p = 0.059) compared with the LF diet (Fig. 5a). On the other hand, the relative abundance of *Verrucomicrobia* showed a sharp increase in the HFC mice. Differing greatly from the HFC diet group, the HFBS diet associated a gut community had a significantly higher relative abundance of *Bacteriodetes* and much lower relative abundance of *Verrucomicrobia*, without a significant change in *Firmicutes*. At the family level, the cecal microbiota of the HFC mice was dominated by *Verrucomicrobiaceae* followed by *Ruminococcaceae* and *Bacteroidaceae*, whereas *Ruminococcaceae* dominated in the LF mice (Fig. 5b). The HFBS diet suppressed the increase in the relative abundance of *Verrucomicrobiaceae* by 85% and induced the relative abundance of *Bacteroidaceae* 5-fold compared with the HFC group, thus leading to the dominance by *Bacteroidaceae*. Increased S24-7 was also observed in the HFBS group compared with the HFC group. At the genus level, the HFC diet leaded to a sharp increase in *Akkermansia* but decreases in *Oscillospira* and *Ruminococcus* (Fig. 5c). The HFBS diet decreased *Akkermansia* by 85% and increased *Bateroides* 5-fold, *Bilophila* 1.67-fold, and *Prevotella* 3.63-fold. Correlation analysis revealed that *Akkermansia (Verrucomicrobia* phylum) was positively correlated with body weight gain, whereas *Bateroides (Bacteroidetes* phylum) was negatively correlated with body weight gain (Supplementary Figure S6). Collectively, these results show that bamboo shoot fiber modulates the gut microbiota of high-fat diet fed mice and results in a microbiota which favors less body weight gain compared with cellulose.

## Discussion

The present study clearly demonstrated the qualitative difference among different types of dietary fibers in their abilities to suppress high-fat diet induced obesity. The insoluble fiber from bamboo shoots performed the best among all tested fibers and the effect was mediated by, at least in part, modulation of the gut microbiota.

Our findings about the difference in controlling body weight among fiber types are consistent with literature. A long-term study on high-fat induced obesity in C57BL/6J mice showed that when supplemented with the same amount, soluble fiber guar gum induced an obesity phenotype, whereas insoluble fiber from oats suppressed obesity^27^. Another soluble fiber, inulin also did not show any difference from cellulose in body weight gain in a mice model of high-fat diet induced obesity^26^. Wheat fiber has long been known to be effective in controlling body weight and reducing cardiovascular and diabetes risks^6^,^7^,^8^,^19^. Recent studies demonstrated that wheat fiber and phytochemicals isolated from wheat bran suppressed body weight gain in high-fat diet fed mice^20^. In agreement with these reports our results showed that insoluble fibers were more effective in suppressing high-fat diet induced obesity than soluble fibers. In addition, bamboo shoot fiber outperformed other insoluble grain fibers. In the context of prevention of obesity, inclusion of soluble fiber should be no more than one third of total fiber content. The reason why we did not observe a significant effect in suppressing body weight of wheat fiber may be due to the short duration of this study. To our knowledge, this is the first demonstration of the anti-obesity effects of insoluble fiber from bamboo shoots.

Given the difference in body weights among the nine groups, we first examined whether difference in energy intake played a role. Surprisingly, energy intake was comparable for the HFC and HFBS groups. Wheat fiber was shown to prevent obesity but with increased energy intake when supplemented to a high-fat diet in mice^9^. In many cases, energy intake was not correlated with fat accumulation^9^,^20^,^31^. Although we did not measure food intake in all the nine groups, it is likely that factors other than food intake play important roles in the difference in controlling body weight among various types of fibers. Next, we measured faecal lipids. We found out that significantly higher amounts of lipids were excreted from feces in the bamboo shoot fiber group (see Supplementary Figure S7). This finding is consistent with the result from *in vitro* test showing that the bamboo shoot fiber has the highest oil-holding capacity compared with cellulose, wheat fiber, and soybean fiber (Supplementary Figure S8). Insoluble hydroxypropyl methylcellulose and wheat fiber were also reported to result in more fecal lipid excretion^20^,^22^. Our results suggest that inclusion of bamboo shoot fiber resulted in the least metabolizable energy without affecting feeding behavior compared with other fibers. Since obesity results from a prolonged energy imbalance, body weight difference may also result from difference in energy expenditure. Although we did not measure energy expenditure, a bigger physical activity was observed for HFBS-fed mice. Dietary fiber varies greatly on chemical and physical properties which will ultimately determine their varied physiological functionality. What discriminates the bamboo shoot fiber from other fibers in its chemical features may explain why bamboo shoot fiber has a greater role in body weight control. However there is very limited information on chemical composition except high insoluble fiber content in bamboo shoot fiber in general^32^. Chemical analysis showed that the insoluble bamboo shoot fiber contains 30.8% cellulose and 41.8% hemicelluloses (Supplementary Table S2). Cellulose is strictly nonfermentable. Hemicelluloses are partially fermentable. The exact composition of the hemicelluloses of the bamboo shoot fiber is largely unknown. Wheat fiber is characterized by arabinoxylan and water-extractable high molecular weight arabinoxylans exhibited prebiotic effects^33^. Soybean fiber is characterized by a high content of oligosaccharides which are highly fermentable and promote weight gain^34^. Inulin is a linear fructose polymer belonging to the group of fructans. It is a typical soluble fiber and efficiently fermented by gut microbes^26^. *In vitro* test showed that bamboo shoot fiber exhibited the highest water and oil holding capacities as compared with cellulose, wheat fiber, and soybean fiber (Supplementary Figure S8), indicating its distinct chemical feature. The structure basis for the extraordinary physical-chemical properties and physiological activities of bamboo shoot fiber remained to be defined.

The risk factors associated with obesity are linked, in part, to the fat-induced hypertrophy and hyperplasia of adipocytes^35^,^36^. The present data showed supplementation of a high-fat diet with bamboo shoot fiber suppressed body weight gain through suppressing hyperplasia of adipocytes. Consistent with reduced fat mass in the HFBS group compared with the HFC group, decreased circulating levels of leptin were observed. No increase in expression of inflammatory genes in the adipose tissue was observed in long term studies^37^,^38^. This could be explained by relatively higher fiber contents in our high fat diets and the short duration of the experiment. Even though the HFC mice showed significant weight gain, they were still in the very early stage of obesity and were relatively healthy compared with the obese mice with elevated inflammatory biomarkers in adipose tissues^37^,^38^. Reduced fasting glucose and insulin levels in mice fed bamboo shoot fiber suggests increased insulin sensitivity. Future study with ITT test could provide direct evidence. Detailed analysis of phosphorylation of Akt and IRS will also help to elucidate the underlying mechanisms of improved insulin sensitivity by bamboo shoot fiber.

The gut microbiota has emerged as an important factor in the development of obesity and metabolic disorders^12^,^13^,^22^,^39^. The interaction between dietary components and intestinal microorganisms shapes the composition of the gut microbiota and thus has a great impact on host metabolism^40^. The cecum was chosen for sampling because previous studies^12^,^41^ showed that it is colonized with sufficient quantities of a readily harvested microbiota for metagenomic analysis. Although a porcine model and analysis of colon microbial ecology would have been much more appropriate from the point of view of colon fermentation^42^,^43^, however, the mice model is wildly adopted for studying gut microbiota based on the fact that the mouse and human microbiota(s) are similar at the division (superkingdom) level, with *Firmicutes* and *Bacteroidetes* dominating^44^. Many studies have shown that increased ratios of *Firmicutes* to *Bacteroides* are associated with the obesity phenotype^44^,^45^. Conversely, increased relative abundance of *Bacteroides* was proportional to the degree of weight loss in obese human subjects^14^. In agreement with these findings, we observed that the relative abundance of *Bacteroidetes* in HFBS mice increased five times compared with that in the HFC mice and was much higher than that in the LF mice, suggesting that bamboo shoot fiber selectively promoted growth of *Bacteroidetes* compared with cellulose. Unique to our study, the sharply increased relative abundance of *Verrucomicrobia* induced by high-fat feeding was suppressed by bamboo shoot fiber. *Verrucomicrobia* have been reported to remain in high level colonization of human gut following extensive antibiotic treatment^46^. Our results showed that extremely high palm oil content in the diet can shift the dominant taxa at division level from *Firmicutes* to *Verrucomicrobia* with little change in *Bacteroidetes*, thus the increase ratio of *Verrucomicrobia* to *Bacteroidetes* can also be associated with the obese phenotype. This is the first evidence of the role of *Verrucomicrobia* in obesity. Bamboo shoot fiber may prove to be useful in correcting not only obesity related but also antibiotic induced gut dysbiosis.

Differences were found not only at division level but also at all lower taxa levels between bamboo shoot fiber and cellulose groups. Several dietary fibers were reported to have prebiotic effects^20^,^21^,^22^,^33^. Chitin-glucan fiber improved host metabolic alterations induced by high-fat diet in mice through increased gram-positive bacteria from clostridial cluster XIVa including *Roseburia*^21^. Wheat fiber counteracted high-fat induced dysbiosis by restoring of *Roseburria* spp. and *Bacteroides/Prevotella* spp.^33^. The 454-pyrosequencing method showed that hydroxypropyl methylcellulose in a high-fat diet induced changes at lower taxonomic levels within the phylum *Firmicutes*^22^. Our results indicate that bamboo shoot fiber induces much more extensive changes in the gut microbiota compared with these fibers, highlighting the need for further characterization of the chemical structure of bamboo shoot fiber and functional consequences of the changed gut microbiota induced by specific fibers.

Modulations in the gut microbiota by bamboo shoot fiber were observed not only in the composition but also in the structure. A high-fat diet with cellulose induced a significant drop in diversity of the gut microbiota, as described previously^22^. However, the same diet with bamboo shoot fiber resulted in much higher diversity, although not at the same level as in the low fat diet group, highlighting that the deleterious effects of high-fat diet on the gut microbiota cannot be completely restored by inclusion of sufficient amount of good quality fiber. The importance of maintaining diversity of the gut microbiota lies in the fact that diet-induced extinctions in the gut microbiota compound over generations^47^. A recent study showed that changes in the microbiota of mice consuming a low fiber diet and harbouring a human microbiota are largely reversible within a single generation. However, a progressive loss of diversity induced by a low fiber diet over several generations is not recoverable. Thus a modern diet low in fiber contributes to the loss of taxa over generations and may explain the lower-diversity microbiota in the populations in the industrialized world compared to rural agrarians^47^. Since bamboo shoot fiber showed better effect in controlling body weight than other fibers, future investigation by using humanized mice models and transfer experiments will elucidate more details in their difference in modulating the gut microbiota.

The significantly increased relative abundance of *Bacteroidetes* and other changes in gut microbiota we observed for bamboo shoot fiber may be explained by the combined effects of high hemicelluloses content and fast transit time. Many factors have been shown to influence fermentability of dietary fiber including solubility, types of linkage, degree of polymerization, and transit time^24^. Soluble dietary fiber is more effectively fermented than insoluble dietary fiber in the gut, and produces abundant SCFAs to regulate gut microbiota balance. Although bamboo shoot fiber is insoluble, it consists of both nonfermentable cellulose and fermentable hemicelluloses. So like grain fibers, bamboo shoot fiber is semi-fermentable. The high cellulose content of bamboo shoot fiber may partially mediate the gut microbiota by increasing fecal bulk and transit time due to complex interactions among diet, gastrointestinal (GI) transit, and gut microbiota. It is shown in humanized mice that although not fermented by human gut microbes, cellulose led to accelerated GI transit and significantly altered gut microbial composition with an increase in the relative abundance of *Bacteroides*^48^.

In conclusion, the present study provided evidence of quality difference of fiber types and proved that the insoluble fiber from bamboo shoots is the most effective in suppressing high-fat diet induced obesity and accompanied changes in metabolism. The suppression of body weight gain was associated with maintaining diversity of the gut microbiota and suppression of high-fat diet induced dysbiosis. Considering six weeks is too short to study bamboo shoot fiber on lipid metabolism and gut microbiota in C57BL/6J mice fed a macronutrient matched high-fat diet, long term feeding study of bamboo shoot fiber should be adopted to validate the findings of this 6 weeks short time study. Further investigations should delineate the mechanisms by which bamboo shoot fiber modulates the microbial ecosystem. Our findings indicate that bamboo shoot fiber may have potential applications in the prevention of obesity and metabolic syndrome.

## Methods

### Plant material, bamboo shoot fiber extraction, and analysis of chemical composition and physicochemical properties chemical

Fresh bamboo shoots of *Dendrocalamus hamiltonii* were collected from Mengla, Yunnan Province, China in August 2014. The botanical authentication was carried out by Professor Youkai Xu at Xishuangbanna Tropical Botanical Garden (XTBG), Chinese Academy of Sciences (CAS). A voucher specimen (HITBC_152673) was deposited at the herbarium of XTBG, CAS. The dried bamboo shoot powders were used to extract dietary fiber as described^49^ with slight modification. In brief, the boiling water treatment in 5 min was used to solubilize and remove sugars. Then, the samples were further concentrated by rinsing until sugar content reached below 0.5% (w/w dry matter). Proximate composition including moisture, protein, lipid, ash and sugar content was determined following the recommendations of the Official Methods of Analysis^50^. Neutral detergent fiber (NDF), acid detergent fiber (ADF) and acid detergent lignin (ADL) were determined as described previously^51^. Soluble and insoluble dietary fibers were determined following AOAC 991.43^50^. Total dietary fiber was calculated by adding IDF and SDF. Physicochemical properties including the water holding capacity (WHC) and the oil holding capacity (OHC) were determined as described previously^52^.

### Ethics statement and experimental design

All animal experimental procedures were approved by the Institutional Animal Care and Use Committee of XTBG, CAS. All the experiments were performed in accordance with the guidelines and regulations of Laboratory Animal Care and Use in China. Eight-week-old female C57BL/6J mice were purchased from Vital River Laboratories (Beijing, China) and housed at a constant temperature of 22 ± 2 °C with a relative humidity of 55 ± 5% and an artificial 12-h light-darkness cycle. After acclimatization to the experimental environment for one week and on a powdered low-fat control diet (LF, AIN-93; 10% energy from palm oil) for 2 weeks before experiment, the mice were randomly assigned to nine groups including one group continued to be on LF diet and eight high-fat diet (HF, AIN-93 adapted; 60% of energy from palm oil) groups supplemented with 10% of eight different dietary fibers (n = 15), respectively (see Supplementary Table S1). Microcrystalline cellulose (Seebio Biotechnology Co. Ltd., Shanghai, China), wheat fiber (Right Wang Biotechnology Co. Ltd., Shanghai, China), soybean fiber (Biotech Co. Ltd. Shanghai, China), and inulin (Ci Yuan Biotechnology Co., Ltd., Xi’an, China) were purchased from manufactures. Three fiber mixes were prepared by mixing bamboo shoot fiber with inulin with ratios of 2:1, 3:1 and 9:1. The gross energy content (571–574 kcal/100 g) and macronutrient composition of the diets (25.2% carbohydrates, 25% protein, 33.5% lipids and 10% dietary fiber) was similar among the HF groups.

During the experiments, body weight and food intake were recorded weekly. Energy intake (kcal) was calculated by multiplying grams of food consumed by the energy (kcal/g) in each diet. Feces were collected weekly and stored at −80 °C. At the end of study, plasma, liver, WAT, cecum content and feces were collected after 12-h-fasting. Parametrial WAT was fixed in 10% neutral formalin, and embedded in paraffin for H&E staining using standard protocols.

### Biochemical analysis

Liver lipid and feces lipid were extracted as described previously^20^. Plasma triglyceride and cholesterol concentrations were determined using the commercially available kits (BioSino, Beijing, China). Plasma insulin concentration was measured using the mouse insulin ELISA kit (Mercodia, Uppsala, Sweden). Leptin was determined using mouse ELISA kit (Jingmei Bio, Jiangsu, China) according to the manufacturer’s instruction.

### Glucose tolerance tests

At week 5 of the experiment, mice were fasted for 6 h. Then the animals were gavaged 1 g/kg body weight of D-glucose solution and tail vail blood was collected at 0, 15, 30, 60, and 120 min. Blood glucose concentrations were measured with a glucose meter (Bayer HealthCare LLC, Mishawaka, USA). Seventy-five micro liters of blood was sampled 15 min before the glucose load to assess plasma insulin concentrations. Homeostasis model assessment of insulin resistance (HOMA-IR) was calculated according to the reported formula^21^.

### Real-time quantitative PCR

Total RNA was extracted from liver and WAT samples using Trizol reagent (Invitrogen, USA) and quantified using Bio-Nano and agarose gel electrophoresis. Complementary DNA (cDNA) was reverse transcribed from 1 μg RNA using PrimeScriptTM RT reagent kit (TaKaRa, Japan). RT-PCR detection of gene expression was performed using a SYBR Premix Ex TaqTM II kit (TaKaRa, Japan) on the LightCycler 480 II system of the fluorescent quantitative PCR (Roche, Switzerland). The thermocycler conditions were: initial denaturation at 95 °C for 30 s, 40 cycles of 95 °C for 10 s, and 58 °C for 1 min. The sequences of the primers (Supplementary Table S3) were synthesized by BGI (Shenzhen, China). The expression of each gene was normalized to the reference gene, β-actin, and expressed relative to the control group according to the 2^−ΔΔCt^ method.

### Microbiota analysis by 16S rRNA gene sequencing

Total genome DNA was extracted from the cecal content using a QIAamp DNA Stool minikit (Qiagen, Hilden, Germany) following the manufacturer’s instructions. Genomic DNA concentration was normalized to 1 ng/μl. The V3-V4 region (~450 bp) of the 16S rRNA gene was amplified using forward primer 341F (5′-CCTACGGGNGGCWGCAG-3′) attached to the Roche B adapter for Illumina library construction and reverse primer 802R (5′-TACNVGGGTATCTAATCC-3′) attached to the Roche A adapter and a 10-nt barcode [5′-A-adapter-N (10) + 16S primer-3′]. PCR reactions were performed as follows: 10 ng of the purified DNA, 15 μl of Phusion^®^ High-Fidelity PCR Master Mix (New England Biolabs), 200 nmol/L of forward and reverse primers, and nuclease-free water in a final volume to 30 μl. PCR cycling condition consisted of an initial denaturation of 1 min at 98 °C, 30 cycles of 10 s at 98 °C, 30 s at 50 °C, and 5 min at 72 °C. PCR products were quantified (400–450 bp), pooled in equimolar ratios, purified (GeneJET Gel Extraction Kit, Thermo Scientific), and used for the Illumina HiSeq/MiSeq platform sequencing to a depth of at least 20,000 reads per sample (mean reads per sample 48,410 ± 984).

Reads were merged by using FLASH^53^ processed using the QIIME (Quantitative Insights Into Microbial Ecology) analysis pipeline^54^. Paired-end joined sequences were grouped into operational taxonomic units (OTUs) using the GreenGenes database^55^ and the UPARSE algorithm^56^ with a 97% threshold of pairwise identity. Samples were grouped according to the diet groups. A representative sequence for each OTU was aligned and the RDP classifier^57^ was used to annotate taxonomic information for each representative sequence. Analysis was performed at each taxonomical level (Phylum, Class, Order, Family, Genus, Species), separately. In-house Perl scripts were used to analyze alpha- (within samples) and beta- (among samples) diversity. Unweighted unifrac for Principal Coordinate Analysis (PCoA) and Unweighted Pair Group Method with Arithmetic mean (UPGMA) Clustering tree were used to assess the variation between experimental groups (beta diversity). Alpha diversity was calculated for all the samples.

### Statistics

All data are presented as the mean ± SEM. The significance of difference among diet groups was analyzed by one-way ANOVA followed by Turkey’s test. A *p*-value < 0.05 was considered to be significant. All analyses were performed using GraphPad Prism version 5.0 (GraphPad Software, San Diego, CA).

## Additional Information

**How to cite this article**: Li, X. *et al*. Bamboo shoot fiber prevents obesity in mice by modulating the gut microbiota. *Sci. Rep.* **6**, 32953; doi: 10.1038/srep32953 (2016).

## Supplementary Material

Supplementary Information

## Figures and Tables

**Table 1:** Effects of bamboo shoot fiber on plasma parameters.

Parameters	LF	HFC	HFBS
Glucose (mmol/L)	3.70 ± 0.15	6.81 ± 1.6^***^	3.86 ± 0.36^**###**^
Insulin (pmol/L)	15.0 ± 1.6	22.8 ± 2.1^*^	12.6 ± 0.7^**###**^
HOMA-IR	2.54 ± 0.08	3.26 ± 0.29^*^	1.99 ± 0.03^**###**^
Leptin (pg/ml)	468.8 ± 28	793.0 ± 27^***^	662.2 ± 9.2^***##^
AUC of GTT	741.6 ± 37.9	1129 ± 73.8^*^	931.5 ± 37.3^*#^
Plasma TG (mmol/L)	0.44 ± 0.01	0.66 ± 0.04^***^	0.56 ± 0.02
Plasma TC (mmol/L)	2.92 ± 0.21	3.64 ± 0.27^*^	2.66 ± 0.10^**##**^
HDL-Cholesterol (mmol/L)	1.03 ± 0.08	1.44 ± 0.11^**^	0.99 ± 0.05^**##**^
LDL-Cholesterol (mmol/L)	1.83 ± 0.06	1.92 ± 0.16	1.47 ± 0.05^**#**^

Data are expressed as mean ± SEM (eight mice for each group). LF, low fat diet; HFC, high-fat diet with cellulose; HFBS, high-fat diet with bamboo shoot fiber. HOMA-IR, homeostasis model assessment of insulin resistance. AUC, area under the curve (0–120 min). *p < 0.05, **p < 0.01, ***p < 0.001, vs. mice fed LF diet. ^**##**^p < 0.01, ^**###**^p < 0.001, vs. mice fed HFC diet.

## References

[b1] Ng M. . Global, regional, and national prevalence of overweight and obesity in children and adults during 1980–2013: a systematic analysis for the Global Burden of Disease Study 2013. Lancet 384, 766–781 (2014). 24880830 10.1016/S0140-6736(14)60460-8 PMC4624264

[b2] Chang C. J. . Ganoderma lucidum reduces obesity in mice by modulating the composition of the gut microbiota. Nat. Commun. 6, 7489 (2015). 26102296 10.1038/ncomms8489 PMC4557287

[b3] Schwiertz A. . Microbiota and SCFA in lean and overweight healthy subjects. Obesity 18, 190–195 (2010). 19498350 10.1038/oby.2009.167

[b4] Barte J. C. . Maintenance of weight loss after lifestyle interventions for overweight and obesity, a systematic review. Obes. Rev. 11, 899–906 (2010). 20345430 10.1111/j.1467-789X.2010.00740.x

[b5] Chandalia M. . Beneficial effects of high dietary fiber intake in patients with type 2 diabetes mellitus. New Engl. J. Med. 342, 1392–1398 (2000). 10805824 10.1056/NEJM200005113421903

[b6] Weickert M. O. What dietary modification best improves insulin sensitivity and why? Clin. Endocrinol. 77, 508–512 (2012). 10.1111/j.1365-2265.2012.04450.x 22640465

[b7] Galisteo M., Duarte J. & Zarzuelo A. Effects of dietary fibers on disturbances clustered in the metabolic syndrome. J. Nutr. Biochem. 19, 71–84 (2008). 17618108 10.1016/j.jnutbio.2007.02.009

[b8] Papathanasopoulos A. & Camilleri M. Dietary fiber supplements: effects in obesity and metabolic syndrome and relationship to gastrointestinal functions. Gastroenterology 138, 65–72 (2010). 19931537 10.1053/j.gastro.2009.11.045 PMC2903728

[b9] Han S. . Dietary fiber prevents obesity-related liver lipotoxicity by modulating sterol-regulatory element binding protein pathway in C57BL/6J mice fed a high-fat/cholesterol diet. Sci. Rep. 5, 15256 (2015). 26510459 10.1038/srep15256 PMC4625144

[b10] Isken F., Klaus S., Osterhoff M., Pfeiffer A. F. & Weickert M. O. Effects of long-term soluble vs. insoluble dietary fiber intake on high-fat diet-induced obesity in C57BL/6J mice. J. Nutr. Biochem. 21, 278–284 (2010). 19369060 10.1016/j.jnutbio.2008.12.012

[b11] O’Hara A. M. & Shanahan F. The gut flora as a forgotten organ. EMBO Rep. 7, 688–693 (2006). 16819463 10.1038/sj.embor.7400731 PMC1500832

[b12] Turnbaugh P. J. . An obesity-associated gut microbiome with increased capacity for energy harvest. Nature 444, 1027–1031 (2006). 17183312 10.1038/nature05414

[b13] Clemente J. C., Ursell L. K., Parfrey L. W. & Knight R. The impact of the gut microbiota on human health: an integrative view. Cell 148, 1258–1270 (2012). 22424233 10.1016/j.cell.2012.01.035 PMC5050011

[b14] Ley R. E., Turnbaugh P. J., Klein S. & Gordon J. I. Microbial ecology - Human gut microbes associated with obesity. Nature 444, 1022–1023 (2006). 17183309 10.1038/4441022a

[b15] Ridaura V. K. . Gut microbiota from twins discordant for obesity modulate metabolism in mice. Science 341, 1241214 (2013). 24009397 10.1126/science.1241214 PMC3829625

[b16] Qin J. . A metagenome-wide association study of gut microbiota in type 2 diabetes. Nature 490, 55–60 (2012). 23023125 10.1038/nature11450

[b17] Turnbaugh P. J., Baeckhed F., Fulton L. & Gordon J. I. Diet-induced obesity is linked to marked but reversible alterations in the mouse distal gut microbiome. Cell Host Microbe. 3, 213–223 (2008). 18407065 10.1016/j.chom.2008.02.015 PMC3687783

[b18] World Health Organization. Diet, nutrition and the prevention of chronic disease. WHO Tech. Rep. Ser. 916, 68–69 (2003). 12768890

[b19] Weickert M. O. & Pfeiffer A. F. H. Metabolic effects of dietary fiber consumption and prevention of diabetes. J. Nutr. 138, 439–442 (2008). 18287346 10.1093/jn/138.3.439

[b20] Oishi K. . Wheat alkylresorcinols suppress high-fat, high-sucrose diet-Induced obesity and glucose intolerance by increasing insulin sensitivity and cholesterol excretion in male mice. J. Nutr. 145, 199–206 (2015). 25644338 10.3945/jn.114.202754

[b21] Neyrinck A. M. . Dietary modulation of clostridial cluster XIVa gut bacteria (*Roseburia* spp.) by chitin-glucan fiber improves host metabolic alterations induced by high-fat diet in mice. J. Nutr. Biochem. 23, 51–59 (2012). 21411304 10.1016/j.jnutbio.2010.10.008

[b22] Cox L. M. . The nonfermentable dietary fiber hydroxypropyl methylcellulose modulates intestinal microbiota. FASEB J. 27, 692–702 (2013). 23154883 10.1096/fj.12-219477 PMC3545536

[b23] Brownlee I. A. The physiological roles of dietary fibre. Food Hydrocolloids 25, 238–250 (2011).

[b24] Raninen K., Lappi J., Mykkanen H. & Poutanen K. Dietary fiber type reflects physiological functionality: comparison of grain fiber, inulin, and polydextrose. Nutr. Rev. 69, 9–21 (2011). 21198631 10.1111/j.1753-4887.2010.00358.x

[b25] Dewulf E. M. . Inulin-type fructans with prebiotic properties counteract GPR43 overexpression and PPARgamma-related adipogenesis in the white adipose tissue of high-fat diet-fed mice. J. Nutr. Biochem. 22, 712–722 (2011). 21115338 10.1016/j.jnutbio.2010.05.009

[b26] Weitkunat K. . Effects of dietary inulin on bacterial growth, short-chain fatty acid production and hepatic lipid metabolism in gnotobiotic mice. J. Nutr. Biochem. 26, 929–937 (2015). 26033744 10.1016/j.jnutbio.2015.03.010

[b27] Isken F., Klaus S., Osterhoff M., Pfeiffer A. F. & Weickert M. O. Effects of long-term soluble vs. insoluble dietary fiber intake on high-fat diet-induced obesity in C57BL/6J mice. J. Nutr. Biochem. 21, 278–284 (2010). 19369060 10.1016/j.jnutbio.2008.12.012

[b28] Nirmala C., Bisht M. S. & Laishram M. Bioactive compounds in bamboo shoots: health benefits and prospects for developing functional foods. Int. J. Food Sci. Tech. Mys. 49, 1425–1431 (2014).

[b29] Yang Y. X. . China food composition. Peking University Medical Press 42 (2015).

[b30] Marlett J. A. Soluble dietary fiber workshop. In: Kritchevsky D., Bonfield C., eds. Dietary Fiber in health and disease, 311–313 (New York, NY: Plenum Press 1997).

[b31] Brockman D. A., Chen X. & Gallaher D. D. High-viscosity dietary fibers reduce adiposity and decrease hepatic steatosis in rats fed a high-fat diet. J. Nutr. 144, 1415–1422 (2014). 24991042 10.3945/jn.114.191577

[b32] Singhal P., Bal L. M., Satya S., Sudhakar P. & Naik S. N. Bamboo shoots: a novel source of nutrition and medicine. Crit. Rev. Food Sci. Nutr. 53, 517–534 (2013). 23391018 10.1080/10408398.2010.531488

[b33] Neyrinck A. M. . Prebiotic effects of wheat arabinoxylan related to the increase in bifidobacteria, Roseburia and Bacteroides/Prevotella in diet-induced obese mice. PLoS One 6, e20944 (2011). 21695273 10.1371/journal.pone.0020944 PMC3111466

[b34] Faber T. A., Dilger R. N., Hopkins A. C., Price N. P. & Fahey G. C. Effects of oligosaccharides in a soybean meal-based diet on fermentative and immune responses in broiler chicks challenged with *Eimeria acervulina*. Poult. Sci. 91, 3132–3140 (2012). 23155023 10.3382/ps.2012-02364

[b35] Kawasaki N., Asada R., Saito A., Kanemoto S. & Imaizumi K. Obesity-induced endoplasmic reticulum stress causes chronic inflammation in adipose tissue. Sci. Rep. 2, 799 (2012). 23150771 10.1038/srep00799 PMC3495279

[b36] Hung S. C., Anderson W. H., Albers D. R., Langhorst M. L. & Young S. A. Effect of hydroxypropyl methylcellulose on obesity and glucose metabolism in a diet-induced obesity mouse model. J Diabetes 3, 158–167 (2011). 21599870 10.1111/j.1753-0407.2011.00118.x

[b37] Lee Y. S. . Inflammation is necessary for long-term but not short-term high-fat diet induced insulin resistance. Diabetes 60, 2474–2483 (2011). 21911747 10.2337/db11-0194 PMC3178297

[b38] Shen W., Gaskins H. R. & McIntosh M. K. Influence of dietary fat on intestinal microbes, inflammation, barrier function and metabolic outcomes. J. Nutr. Biochem. 25, 270–280 (2014). 24355793 10.1016/j.jnutbio.2013.09.009

[b39] Everard A. . Responses of gut microbiota and glucose and lipid metabolism to prebiotics in genetic obese and diet-induced leptin-resistant mice. Diabetes 60, 2775–2786 (2011). 21933985 10.2337/db11-0227 PMC3198091

[b40] O’Connor E. M., O’Herlihy E. A. & O’Toole P. W. Gut microbiota in older subjects: variation, health consequences and dietary intervention prospects. Proc. Nutr. Soc. 73, 441–451 (2014). 24824449 10.1017/S0029665114000597

[b41] Backhed F. . The gut microbiota as an environmental factor that regulates fat storage. Proc. Natl. Acad. Sci. USA 101, 15718–15723 (2004). 15505215 10.1073/pnas.0407076101 PMC524219

[b42] Aumiller T., Mosenthin R. & Weiss E. Potential of cereal grains and grain legumes in modulating pigs' intestinal microbiota–A review. Livest. Sci. 172, 16–32 (2015).

[b43] Sun Y., Su Y. & Zhu W. Microbiome-metabolome responses in the cecum and colon of pig to a high resistant starch diet. Front. Microbiol. 7, 779 (2016). 27303373 10.3389/fmicb.2016.00779 PMC4880592

[b44] Ley R. E. . Obesity alters gut microbial ecology. Proc. Natl. Acad. Sci. USA 102, 11070–11075 (2005). 16033867 10.1073/pnas.0504978102 PMC1176910

[b45] Moschen A. R., Wieser V. & Tilg H. Dietary factors: major regulators of the gut’s microbiota. Gut Liver 6, 411–416 (2012). 23170142 10.5009/gnl.2012.6.4.411 PMC3493718

[b46] Dubourg G. . High-level colonisation of the human gut by Verrucomicrobia following broad-spectrum antibiotic treatment. Int. J. Antimicrob. Ag. 41, 149–155 (2013). 10.1016/j.ijantimicag.2012.10.012 23294932

[b47] Sonnenburg E. D. . Diet-induced extinctions in the gut microbiota compound over generations. Nature 529, 212–215 (2016). 26762459 10.1038/nature16504 PMC4850918

[b48] Kashyap P. C. . Complex interactions among diet, gastrointestinal transit, and gut microbiota in humanized mice. Gastroenterology 144, 967–977 (2013). 23380084 10.1053/j.gastro.2013.01.047 PMC3890323

[b49] Elleuch M. . Date flesh: chemical composition and characteristics of the dietary fibre. Food Chem. 111, 676–682 (2008).

[b50] AOAC. Official methods of analysis (17th ed.). Gaithersburg, MD: Association of Official Analytical Chemists (2000).

[b51] Vansoest P. J., Robertson J. B. & Lewis B. A. Methods for dietary fiber, neutral detergent fiber, and nonstarch polysaccharides in relation to animal nutrition. J. Dairy Sci. 74, 3583–3597 (1991). 1660498 10.3168/jds.S0022-0302(91)78551-2

[b52] Mateos-Aparicio I., Redondo-Cuenca A. & Villanueva-Suárez M. J. Isolation and characterisation of cell wall polysaccharides from legume by-products: Okara (soymilk residue), pea pod and broad bean pod. Food Chem. 122, 339–345 (2010).

[b53] Magoc T. & Salzberg S. L. FLASH: fast length adjustment of short reads to improve genome assemblies. Bioinformatics 27, 2957–2963 (2011). 21903629 10.1093/bioinformatics/btr507 PMC3198573

[b54] Caporaso J. G. . QIIME allows analysis of high-throughput community sequencing data. Nat. Methods 7, 335–336 (2010). 20383131 10.1038/nmeth.f.303 PMC3156573

[b55] DeSantis T. Z. . Greengenes, a chimera-checked 16S rRNA gene database and workbench compatible with ARB. Appl. Environ. Microbiol. 72, 5069–5072 (2006). 16820507 10.1128/AEM.03006-05 PMC1489311

[b56] Edgar R. C. UPARSE: highly accurate OTU sequences from microbial amplicon reads. Nat. Methods 10, 996–998 (2013). 23955772 10.1038/nmeth.2604

[b57] Cole J. R. . Ribosomal Database Project: data and tools for high throughput rRNA analysis. Nucleic Acids Res. 42, D633–D642 (2014). 24288368 10.1093/nar/gkt1244 PMC3965039

