# Supplementary material for: Bamboo shoot fiber prevents obesity in mice by modulating the gut microbiota
Source: Sci Rep. 2016 Sep 7;6:32953. doi: 10.1038/srep32953 (PMC5013436; doi:10.1038/srep32953)
Supplement: Supplementary Information [file srep32953-s1.pdf]

Supplementary Information

Bamboo shoot fiber prevents obesity in mice by modulating the gut microbiota

Xiufen Li[1,2], Juan Guo[1], Kailong Ji[1,2] , and Ping Zhang[1,*]

[1]*Key Laboratory of Tropical Plant Resources and Sustainable Use, Xishuangbanna Tropical Botanical Garden, Chinese Academy of Sciences, Menglun, Mengla, Yunnan 666303, China*

[2]*University of Chinese Academy of Sciences, Beijing 100049, China*

Affiliation

*Corresponding author

Ping Zhang, PhD, Key Laboratory of Tropical Plant Resources and Sustainable Use, Xishuangbanna Tropical Botanical Garden, Chinese Academy of Sciences, Menglun, Mengla, Yunnan 666303, PR China

Tel.: +86 691 871 3169; Fax: +86 691 871 3061.

E-mail address: zhangping@xtbg.org.cn

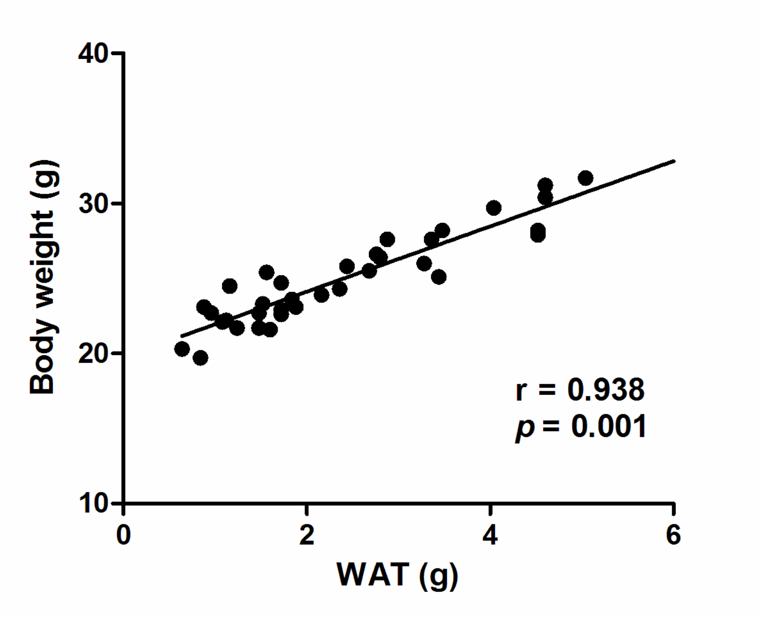

Supplementary Figure S1. Correlation between body weight and white adipose tissue (WAT) ($p < 0.01$) was calculated by Pearson’s r correlation.

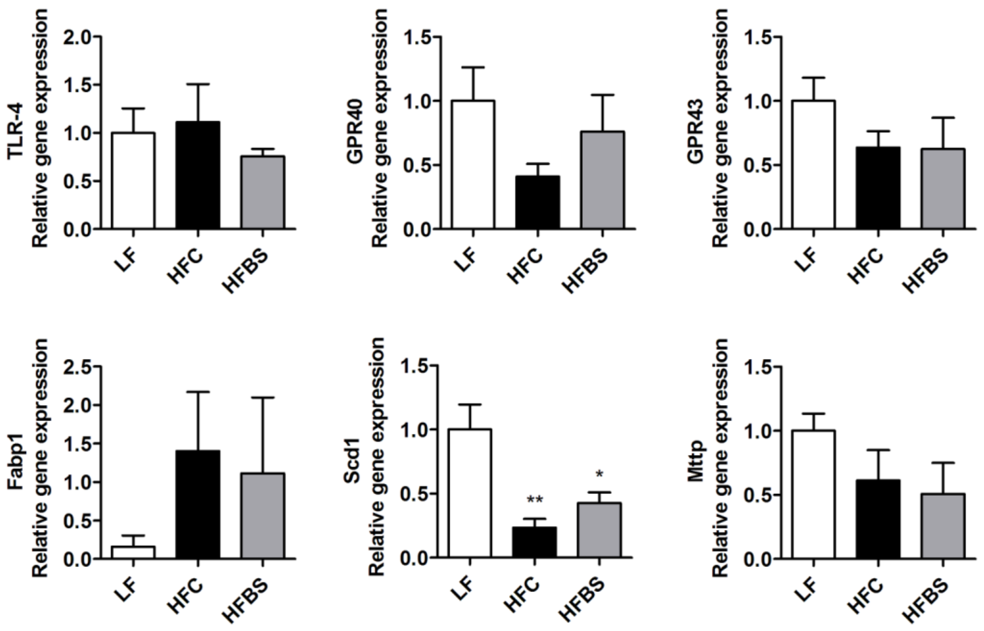

Supplementary Figure S2. The gene expression in adipose tissue. Data are expressed as mean ± SEM (eight mice for each group). LF, low fat diet; HFC, high-fat diet with cellulose; HFBS, high-fat diet with bamboo shoot fiber. *****$p < 0.05$, ******$p < 0.01$, vs. mice fed LF diet.

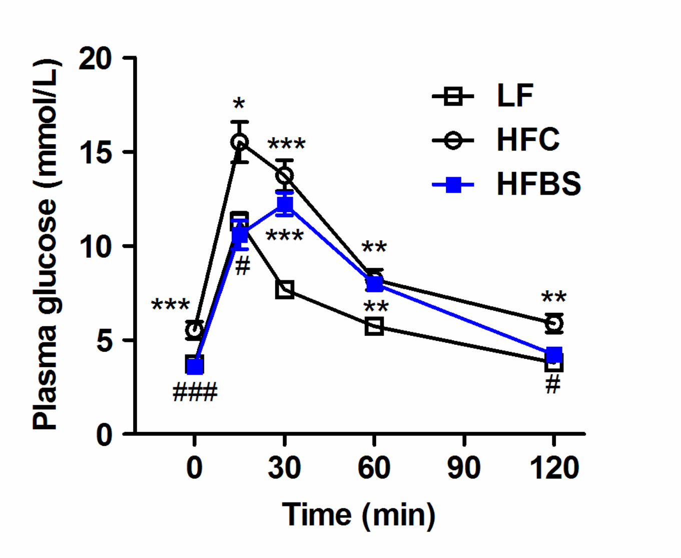

Supplementary Figure S3. Glucose tolerance test. Data are expressed as mean ± SEM (eight mice for each group). LF, low fat diet; HFC, high-fat diet with cellulose; HFBS, high-fat diet with bamboo shoot fiber. *$p < 0.05$, **$p < 0.01$, ***$p < 0.001$, vs. mice fed LF diet. #$p < 0.05$, ###$p < 0.001$, vs. mice fed HFC diet.

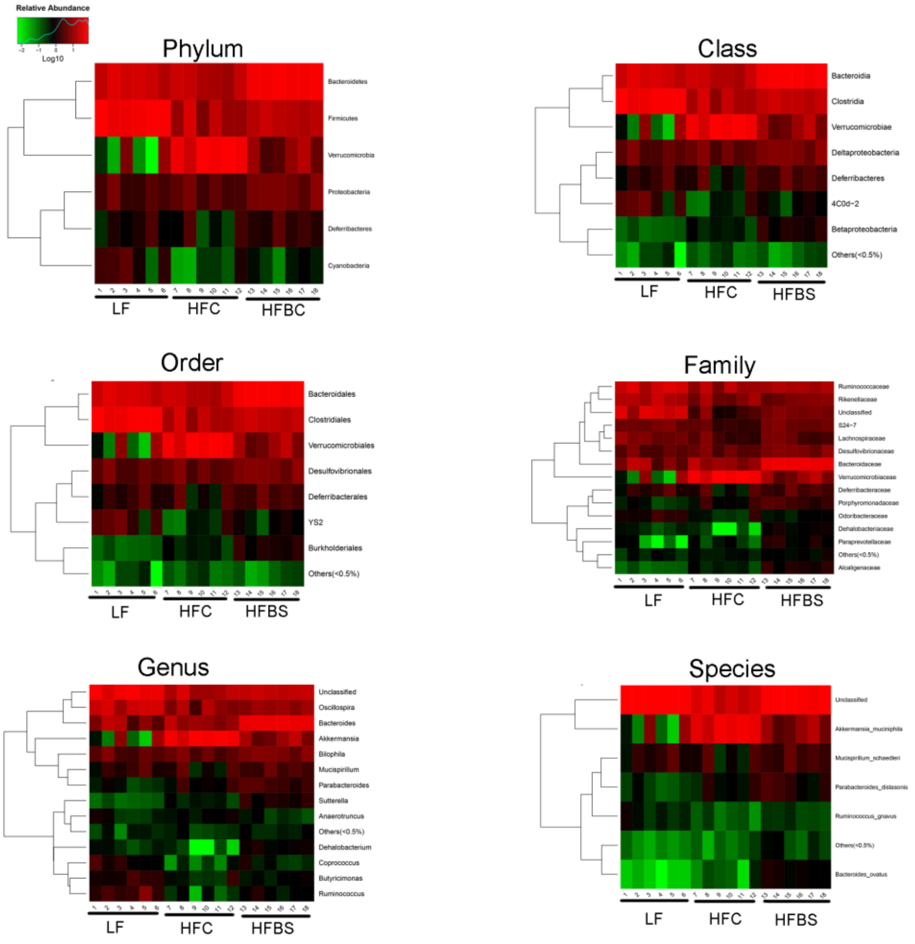

Supplementary Figure S4. Log-scaled percentage heat map of Phylum-level, Class-level, Order-level, Family-level, Genus-level, and Species-level. Data expressed as eight mice for each group. LF, low fat diet; HFC, high-fat diet with cellulose; HFBS, high-fat diet with bamboo shoot fiber.

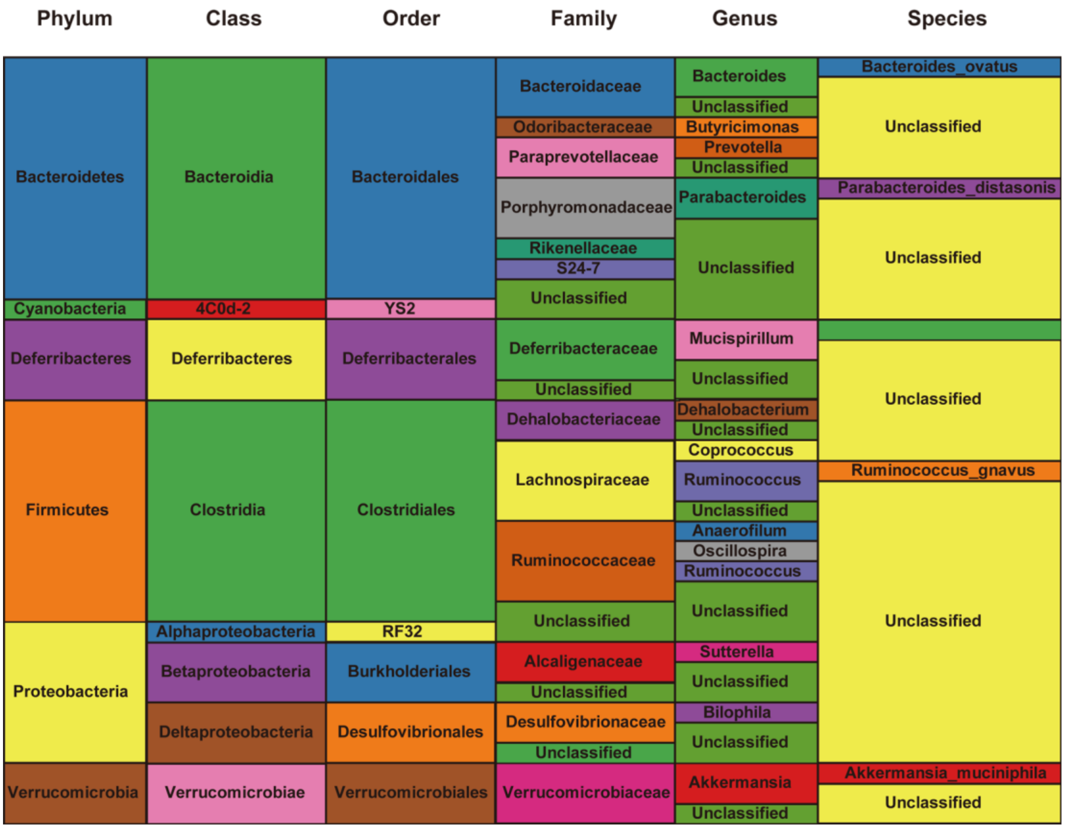

Supplementary Figure S5. Represented bacterial taxa information.

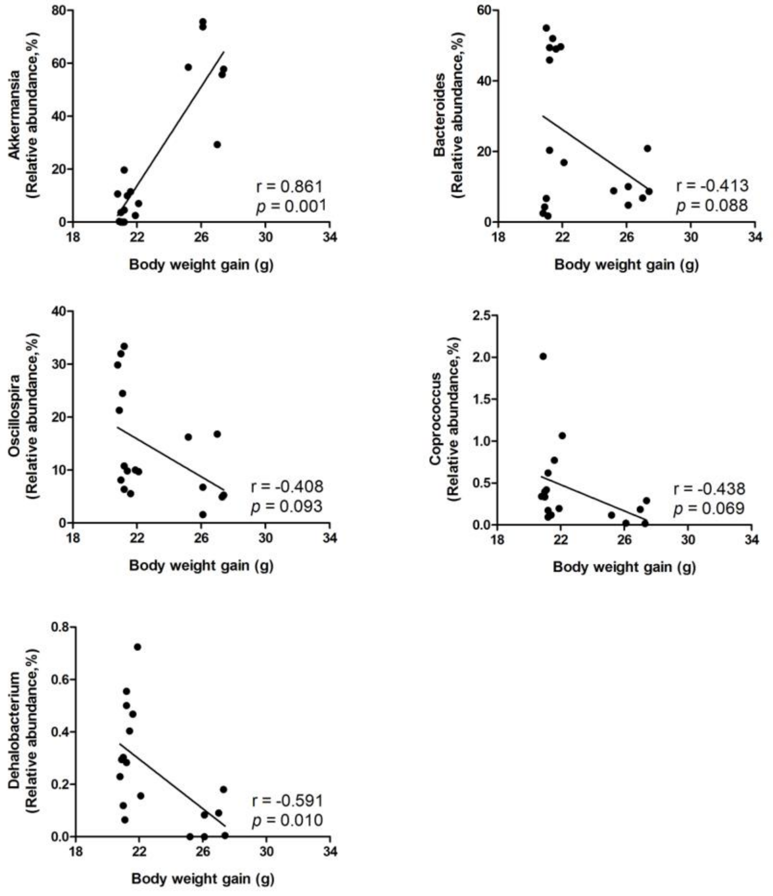

Supplementary Figure S6. Correlation between body weight gain and the relative abundance of (**A**) Akkermansia, (**B**) Bacteroides, (**C**) Oscillospira, (**D**) Coprococcus and (**E**) Dehalobacterium ($p < 0.10$) was calculated by Pearson's r correlation.

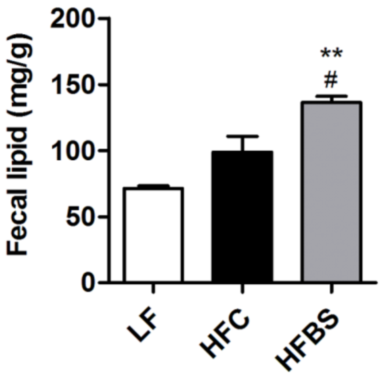

Supplementary Figure S7. Fecal lipid content. Data are expressed as mean ± SEM (eight mice for each group). LF, low fat diet; HFC, high-fat diet with cellulose; HFBS, high-fat diet with bamboo shoot fiber. ******$p < 0.01$, vs. mice fed LF diet, $^{\#}p < 0.05$, vs. mice fed HFC diet.

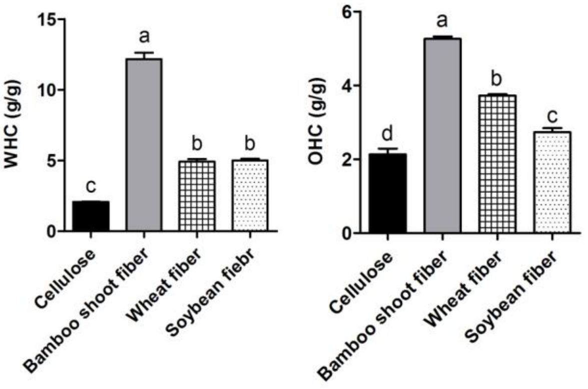

Supplementary Figure S8. Water holding capacity (WHC) and Oil holding capacity (OHC) of dietary fibers tested in this study. Data are expressed as mean ± standard deviation of three replicates. Values designated with different letters are significantly different, $p < 0.05$.

Supplementary Table S1. Composition of the experimental diets. LF, low fat diet; High-fat diets with 10% fiber: cellulose (HFC), wheat fiber (HFW), soybean fiber (HFS), inulin (HFI), bamboo shoot fiber (HFBS), and mixed bamboo shoot fiber and inulin with ratios of 2:1 (HFBSI33), 3:1 (HFBSI25), and 9:1 (HFBSI10).

**Ingredients (g/kg)**	**LF**	**HFC**	**HFW**	**HFS**	**HFI**	**HFBS**	**HFBS I10**	**HFBS I25**	**HFBS I33**
Casein	200	250	250	250	250	250	250	250	250
Corn starch	555	151.6	151.6	151.6	151.6	151.6	151.6	151.6	151.6
Sucrose	100	100	100	100	100	100	100	100	100
Palm oil	44	335	335	335	335	335	335	335	335
Microcrystalline Cellulose	50	100	-	-	-	-	-	-	-
Wheat fiber	-	-	100	-	-	-	-	-	-
Soybean fiber	-	-	-	100	-	-	-	-	-
Inulin	-	-	-	-	100	-	10	25	33
Shoot fiber	-	-	-	-	-	100	90	75	67
AIN-76 mineral mix	35	35	35	35	35	35	35	35	35
AIN-76 vitamin mix	10	10	10	10	10	10	10	10	10
DL-methionine	3	3	3	3	3	3	3	3	3
Choline bitartrate	2	2	2	2	2	2	2	2	2
TBHQ	1	0.4	0.4	0.4	0.4	0.4	0.4	0.4	0.4
Cholesterol	-	10	10	10	10	10	10	10	10
Sodium cholate	-	3	3	3	3	3	3	3	3

Supplementary Table S2. Chemical composition of the isolated dietary fiber from bamboo shoots (*Dendrocalamus hamiltonii*) (% dry matter). Data are expressed as mean ± standard deviation of three determinations.

**Composition**	**%**
Proximate composition	
Total dietary fiber (TDF)	74.5 ± 1. 18
Insoluble dietary fiber (IDF)	73.4 ± 0.89
Soluble dietary fiber (SDF)	1.12 ± 0.29
Protein	18.7 ± 0.47
Lipid	2.75 ± 0.15
Ash	0.70 ± 0.04
Soluble sugars	0.31 ± 0.02
Fiber composition	
Neutral detergent fiber (NDF)	73.6 ± 1.01
Acid detergent fiber (ADF)	31.8 ± 0.91
ADL (Lignin)	0.94 ± 0. 08
Hemicelluose (NDF-ADF)	41.84 ± 0.10
Cellulose (ADF-ADL)	30.83 ± 0.99

Supplementary Table S3. Primer sequence used for real-time PCR.

Gene	5' Primer	3' Primer
PPARα	CAG TGC CCT GAA CAT CGA GTG T	TTC GCC GAA AGA AGC CCT T
Mttp	ATA CAA GCT CAC GTA CTC CAC T	TCC ACA GTA ACA CAA CGT CCA
Fabp1	AAA GGA AAC CTC ATT GCC ACC A	AAT GTC GCC CAA TGT CAT GGT A
Scd1	CCG GAG ACC CTT AGA TCG A	TAG CCT GTA AAA GAT TTC TGC AAA CC
TLR-4	GAAACGGCAACTTGGACCTG	TTCTTTTCCCGAGTTAGGTA
GPR40	TGG CTA GTT TCA TAA ACC CGG	TCC CAA GTA GCC ATG GAC CAG T
GPR43	TGT TCA GTT CCC TCA ATG CCA	CAG GAT TGC GGA TCA GTA GCA
β-actin	TGTCCACCTTCCAGCAGATGT	AGCTCAGTAACAGTCCGCCTAGA
